# Mouse mammary tumor virus (MMTV) - like exogenous sequences are associated with sporadic but not hereditary human breast carcinoma

**DOI:** 10.18632/aging.102252

**Published:** 2019-09-13

**Authors:** Antonio Giuseppe Naccarato, Francesca Lessi, Katia Zavaglia, Cristian Scatena, Mohammad A. Al Hamad, Paolo Aretini, Michele Menicagli, Manuela Roncella, Matteo Ghilli, Maria Adelaide Caligo, Chiara Maria Mazzanti, Generoso Bevilacqua

**Affiliations:** 1 Division of Pathology, Department of Translational Research and New Technologies in Medicine, University of Pisa, and Department of Laboratory Medicine, Pisa University Hospital, Pisa, Italy; 2 Department of Laboratory Medicine, Pisa University Hospital, Pisa, Italy; 3 Fondazione Pisana per la Scienza, Pisa, Italy; 4 Division of Molecular Genetics, Department of Laboratory Medicine, Pisa University Hospital, Pisa, Italy; 5 Department of Pathology, College of Medicine, Imam Abdulrahman Bin Faisal University, Dammam, Saudi Arabia; 6 Division of Surgery, Breast Center, Pisa University Hospital, Pisa, Italy; 7 “San Rossore” Hospital – Casa di Cura “San Rossore”, Pisa, Italy

**Keywords:** MMTV, HMTV, sporadic breast cancer, hereditary breast cancer, viral cancer, breast cancer etiology

## Abstract

The inheritance of mutated suppressor genes, such as BRCA1 and BRCA2, is acknowledged as an etiological factor in hereditary breast carcinoma (HBC). Two different molecular mechanisms are possible; the Knudson’s “two hits” or the gene haploinsufficiency. Etiology of sporadic breast carcinoma (SBC) is not known, although data support the possible role of the betaretrovirus Mouse Mammary Tumor Virus (MMTV). This study analyzes the presence of MMTV exogenous sequences in two representative groups of HBC and SBC, excluding any contamination by murine and retroviral material and endogenous betaretroviruses. The 30.3% of 56 SBC contained MMTV sequences, against the 4.2% of 47 HBC (*p* < 0.001). Cases positive for viral sequences showed the presence of p14, signal peptide of the MMTV envelope precursor. This result was expected based on the fact that HBCs, having a specific genetic etiology, do not need the action of a carcinogenetic viral agent. Moreover, the striking results obtained by comparing two groups of vastly different tumors represent an additional element of quality control: the distinction between HBC and SBC is so well-defined that results cannot be ascribed to mere coincidence. This paper strengthens the hypothesis for a viral etiology for human sporadic breast carcinoma.

## INTRODUCTION

Human breast carcinoma (BC) is divided into two wide groups, sporadic (SBC) and hereditary (HBC), each characterized by distinctly different biomolecular pathways and clinical behavior.

The etiology of SBC remains unknown, despite its high frequency (approximately 90% of all cases of BC) and the decades of extensive studies performed worldwide. Recent evidence indicates a close relationship between SBC and the mouse mammary tumor virus (MMTV) [1], a betaretrovirus recognized as the etiological agent of murine mammary tumors [2]. SBC and murine mammary tumors have a high biological and morphological similarity, and our understanding of the pathogenesis of SBC, in particular the concepts of cancer progression, preinvasive lesions, and the promotional role of estrogens, derives from the murine model [2]. In 1995, exogenous MMTV *env*-like sequences (MMTV*els*) were detected in about 40% of infiltrating human SBC [3].

HBCs represent 5-10% of all cases of breast carcinoma. They are induced by highly penetrant pathogenic mutations affecting a group of tumor suppressor genes (TSG), transmitted in an autosomal dominant way from one parent. Two TSGs, BRCA1 and BRCA2 (BReast CAncer), are responsible for 80-90% of cases of “single gene” hereditary breast carcinoma. Women with a mutation in the BRCA1 face as high as 80% lifetime risk of developing breast cancer [4]. According to Knudson's model [5], both alleles of a TSG conferring susceptibility to breast cancer (as BRCA1) need to be mutated to initiate carcinogenesis. One allele is inherited already mutated (first hit), whereas the second one is mutated during the lifetime (second hit). However, there are cases in which the second mutation cannot be demonstrated. Recently, to explain this discrepancy, a great deal of attention has been given to the status of the protein coded by TSG. This applies to hereditary tumors the concept of haploinsufficiency, which is a known causative mechanism of non-neoplastic diseases [6]. Haploinsufficiency occurs when one allele of a gene is inactivated and the remaining functional allele cannot produce protein in a manner that preserves the physiological status. One possible mechanism is that the mutated allele is dominant negative such that the mutant protein interferes with the wild-type protein. The result is similar to the loss of heterozygosity (LOH) caused by the Knudson’s hypothesis, without requiring the inactivation of the second allele. Several studies support the hypothesis that BRCA1 two-hit mechanism is not the only mechanism, and haploinsufficiency is involved [7]. Moreover, BRCA1 haploinsufficiency is unique to normal human breast epithelium, explaining why neoplastic transformation in hereditary breast tumors is limited to the mammary gland in most cases [8, 9]. In summary, the inherited mutated TSG can be considered an etiological factor for HBC.

Based on this, our working hypothesis was the following: if MMTV is the possible etiological agent of SBC, HBC will test negative for MMTV sequences, as they have a specific genetic etiology, and do not need the action of a carcinogenetic viral agent. Therefore, two representative groups of SBC and HBC were analyzed for the presence of MMTV*els*, and the results confirmed that viral sequences are present in a high percentage of SBC, whereas they are almost absent in HBC. Moreover, cases positive for viral sequences were also positive for the presence of the p14 protein, the signal peptide of the MMTV envelope precursor [10]. In addition to the laboratory’s precautions, the highly significant difference between the two groups excludes the possibility of contamination and strengthens the association between MMTV and SBCs.

## RESULTS

Two different groups of infiltrating breast carcinoma were analyzed for MMTV*els*, 47 HBC and 56 SBC. All tumors were classified as no special type. Moreover, in all of them, the presence of the MMTV p14 protein was investigated by immunohistochemistry (IHC).

### All patients with HBC hosted a BRCA germline mutation

All 47 patients with HBC were less than 40 years old and had a germline mutation in a BRCA gene; 25 in the BRCA1 gene and 22 in the BRCA2 gene. The genetic characteristics of all the cases are shown in Table 1. At the same time, wild type BRCA genes were found in the 56 SBC patients, who were all older than 45 years.

**Table 1:** BRCA1 and BRCA2 gene germline mutations in 47 HBC.

**BRCA1**				**BRCA1BRCA2**	
	**mutation type**	**no. of cases**		**mutation type**	**no. of cases**
**MMTV*els* positive cases:**	5154del5 *frameshift*	1		9326insA *frameshift*	1
					
**MMTV*els* negative cases:**	1499insA *frameshift*	4		9326insA *frameshift*	3
	5154del5 *frameshift*	3		E97X *stop*	3
	5382insC *frameshift*	3		c.9253dupA *frameshift*	2
	1100delAT *frameshift*	2		8474delAG *frameshift*	2
	4873delCA *frameshift*	2		ivs13-2at>a *splicing*	1
	Q1395X *stop*	2		6696delTC *frameshift*	1
	3598del10 *frameshift*	2		S1970X *stop*	1
	5035_39del5 *frameshift*	1		W2586X *stop*	1
	del20 *deletion*	1		6954delT *frameshift*	1
	delex14-19 *deletion*	1		1466delT *frameshift*	1
	3875del4 *frameshift*	1		IVS7-2 *splicing*	1
	A1789T *missense*	1		999delTCAAA *frameshift*	1
	3403delA *frameshift*	1		5721_5722delCT *frameshift*	1
				8475delAG *frameshift*	1
				Leu583X *stop*	1
Total number of cases		25			22

HBC: hereditary breast carcinoma.

### MMTV*els* are present in SBC but not in HBC

**Figure 1:**
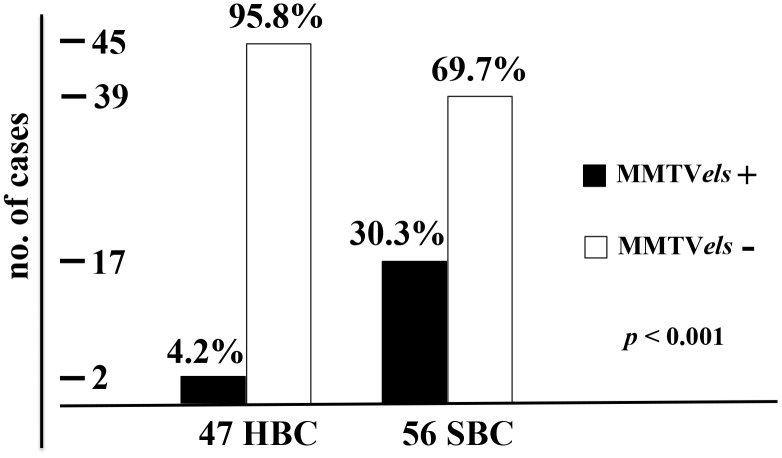
**Frequency of MMTV*els* in 47 HBC and 56 SBC.** HBC cases are positive for MMTV*els* only in 4.2%, whereas positive SBC cases are 30.3%, with a *p* < 0.001. HBC: hereditary breast carcinoma. SBC: sporadic breast carcinomas. MMTV*els*: MMTV *env*-like sequence.

The presence of MMTV*els* in sporadic breast cancer was significantly higher than in hereditary breast cancer (30.3% vs 4.2%, with a *p* < 0.001; Figure 1). In the case of SBCs, 17 (30.3%) of the 56 tumors examined were positive for MMTV*els*, whereas for HBCs only 2 (4.2%) of the 47 tumors examined harbored the viral sequence. One of the two patients carried the BRCA1 gene mutation 5154del5, whereas the other carried the BRCA2 gene mutation 932insA (Table 1).

### MMTV p14 protein is present in MMTV*els*-positive tumors but absent in MMTV*els*-negative tumors

**Figure 2:**
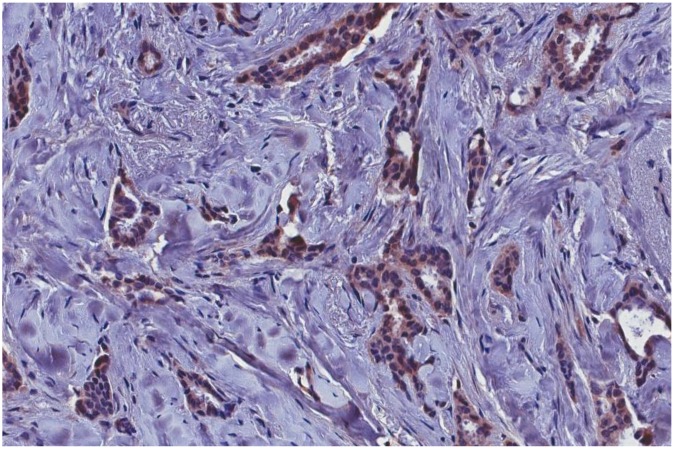
**MMTV*els*-positive infiltrating breast carcinoma cells positive for p-14 protein with immunohistochemical analysis.** MMTV*els*: MMTV *env*-like sequence.

**Figure 3:**
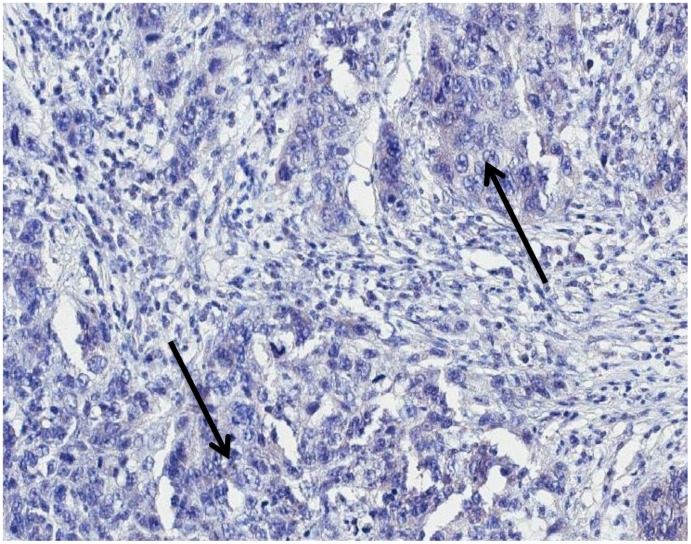
**MMTV*els* negative infiltrating breast carcinoma cells negative for p-14 protein with immunohistochemical analysis (arrows: groups of cancer cells).** MMTV*els*: MMTV *env*-like sequence.

The immunohistochemical analysis showed the presence of p14 protein in all MMTV*els* positive tumors, whereas all negative MMTV*els* tumors were negative for p14 (Figures 2, 3).

### MMTV sequence analysis

The PCR products of the two MMTV*els* identified in the HBC and two of those identified in MMTV*els*-positive SBC were sequenced. The sequence was 100% identical to the reference HMTV sequence (GenBank accession no. AF243039).

### The MMTV*els* sequences are not an artifact of contamination

A possible mouse DNA contamination of MMTV*els*-positive samples was excluded by performing murine mitochondrial DNA and Intracisternal A Particle (IAP) LTRs PCR [11]. All tested samples were found to be free of mouse DNA. Furthermore, the murine housekeeping gene GAPDH was not detected, whereas human GAPDH was amplified.

## DISCUSSION

### HBC has a specific etiological mechanism whereas SBC does not

Inheritance of a mutated TSG can be considered an etiological factor for HBCs. The distinction between the concepts of hereditary and familial deserves some attention. A hereditary event is usually familial, whereas a familial event is not necessarily hereditary. For instance, because obesity is an important risk factor for SBC, a high incidence of breast cancer can be observed in a family in which several members are obese owing to eating habits, in the absence of a hereditary trait. For this reason, in this study, only tumors belonging to women with a BRCA gene germline mutation were considered. A 2010 paper reports a high incidence of MMTV positive cases among “familial breast cancer”; however, they were unpublished, and there is no information about the possible hereditary nature of their familiarity [12].

In contrast, no etiological factor is acknowledged for SBC. A transforming role of estrogens cannot be demonstrated, whereas their relevant pathogenic role is well known [13]. MMTV has been a candidate since the seventies [14], even if only twenty years later, exogenous MMTV*els* were identified in a high percentage of HBCs [3]. Recent evidence indicates close relationship between MMTV and SBC, wherein: a) MMTV*els* were detected in a high percentage of pre- invasive SBC lesions, mainly ductal carcinoma *in situ* (80% of cases) [15]; b) primary cultures of HBC can produce MMTV-like particles [16]; c) MMTV is able to infect *in vitro* human cells of different types including breast cells [17–19], leading to a rapid spread of the virus [20]; e) polypyrimidine tract-binding (PTB) protein, involved in maintaining human breast cancer cell growth and malignant properties, is able to bind the 5’ untranslated region of MMTV mRNA and to stimulate cap-independent translation initiation [21, 22]; f) MMTV*els* were identified in breast tissues prior to the development of MMTV*els*-positive breast cancer [23]; and g) MMTV *env* sequences are absent in the human genome, whereas present in breast tumors and in normal breast tissues [24]. Finally, MMTV sequences have been detected in human salivary glands and saliva, suggesting saliva as a possible route of inter-human spread of MMTV [25]. MMTV could operate by the classic mechanism of insertional mutagenesis, which takes place in murine mammary tumors. Recently, it has been shown that the overexpression of WNT1 and FGF3, the two main integration sites of MMTV in mice, increases mammosphere formation and promotes stem cell activity in human MCF7 cells [26]. The difficulty in detecting viral DNA without using PCR- and nested PCR-based techniques is not in favor of the hypothesis of the chromosomal insertion of MMTV. However, the fact that MMTV*els* are present in more than 80% of ductal carcinoma *in situ* against the 30-40% of infiltrating cancer indicates that the virus could be relevant for cell transformation only and not for cancer progression. The 50% reduction of positive cases moving from *in situ* to infiltrating lesions can be a consequence of DNA loss owing to the high level of chromosomal rearrangement characterizing breast tumors. Furthermore, chromogenic *in situ* hybridization experiments have demonstrated the presence of viral hybridization signal in tumor nuclei, with its strong reduction in infiltrating tumors when compared to carcinoma *in situ* [15].

Interestingly, the MMTV envelope protein seems to be directly involved in oncogenic transformation; in fact, MMTV *env* encodes an immunoreceptor tyrosine-based activation motif (ITAM) responsible for the transformation of human mammary epithelial cells in culture [27]. Again, other viruses have been associated to BC, such as Bovine Leukemia Virus (BVL), Human Papilloma Virus (HPV), and Epstein-Barr Virus (EBV) [28, 29], even if, differently from MMTV, there is no experimental model for any of them. In any case, MMTV could exert its oncogenic action through the secondary activation of one of them. Possible non-viral carcinogenetic agents are not known, except the very few cases due to radiations.

### MMTV*els* are associated with SBC but not to HBC

This study confirms the presence of MMTV sequences in 30% of SBC, a percentage consistent with that reported in previous papers, and demonstrates their almost absence in HBC, with almost 96% of negative cases. The difference between the two groups is highly significant, with a *p*-value < 0.001.

In 2011 Park et al. [30] reported the absence of MMTV*els* in a series of 42 invasive breast carcinomas, interpreting their negative result hypothesizing that the positive cases reported in the literature were all due to PCR contamination. Pogo and colleagues [31] immediately commented on this paper, demonstrating that the methodology used was unable to proficiently amplify MMTV*els* gene sequences in breast cancer DNA. However, focusing on the aim of the present paper, it is interesting to note that in Park’s article all the patients were enrolled in the Australian Breast Cancer Family Study, and all of them received a diagnosis of cancer before the age of 40. With these data, we can hypothesize that all these cases are presumably HBC, hereditary breast carcinoma, and therefore MMTV negative, which would reinforce our results. The only two HBC cases that we found positive for MMTV*els* are due with high probability to mere chance.

In contrast, a paper of 2008 [32] describes the presence of MMTV-like sequences in breast tumors occurring in three members of the same family (father, mother, daughter). The age of the patients (> 50 years) and, primarily, the fact that two of them (father and mother) were genetically unrelated, indicate that the authors were dealing with sporadic neoplasms. Interestingly, this report, together with that of Pogo et al of 2010 (12), suggests the possibility of MMTV as an environmental etiological agent.

### MMTV p14 protein is present in MMTV associated SBCs while absent in HBCs

P14, a multifunctional 98 amino acid peptide, is the signal peptide of the MMTV envelope precursor, localized in the nucleolus of cells harboring the virus with nucleo-cytoplasmic shuttling [10]. It is a tumor modulator phosphoprotein, endogenously phosphorylated by two serine kinases, CK2 at serine 65 and PKC at serine 18. P14 functions as an oncogene when serine 65 is phosphorylated, while it functions as an anti-oncogene when serine 18 is phosphorylated [10]. It is expressed on the surface of MMTV-associated murine and human cells [33]. P14 was immunohistochemically detected in all cases positive for MMTV*els*, whereas was absent in all negative cases. This result strengthens the molecular data.

### MMTV sequences in SBCs are not due to contamination

The possibility that positive MMTV results were a consequence of contamination has become a recurring motif [34], even if with dubious and inconclusive positions: their positive “results were not consistent and seemed to be an artifact”, “experiments indicated that the probable source of false positives was murine DNA … present in our building”, and “…published data, indicates that there are some very unique human MMTV sequences in the literature”. Unfortunately, these skeptical papers are so focused on the issue of contamination that they forget to comment on the numerous biological and molecular data that link MMTV to human breast cancer.

Regarding the present study:

- all SBC and HBC paraffin blocks come from the same archive, and were processed in the same laboratory with the same equipment and reagents,
- for the molecular analysis tissues were processed and analyzed all together in the same laboratory,
- the laboratory in which the analyses were conducted does not host mice nor murine cells,
- the difference in results between SBC (with a high percentage of positive cases for MMTV sequences: 30.3%) and HBC (with a very high percentage of negative cases: almost 96%) is too distinct (*p* < 0.001) for it to be a mere coincidence,
- the p14 IHC results match perfectly with the molecular data: all MMTV*els*-positive cases were positive at the immunohistochemical analysis, whereas all MMTV*els*-negative cases were negative,
- the percentage of SBCs positive for MMTV sequences (30.3%) is consistent with the results of previous studies.

### MMTV sequences analyzed are of exogenous origin

Another critical point is represented by the possibility that MMTV sequences identified in human tissues belong to endogenous betaretroviruses. In this case, their infectious nature would be excluded.

The MMTV sequences investigated in this paper were already demonstrated to be exogenous in the original papers by Pogo’s laboratory [3, 35, 36]. Moreover, a previous paper [15] by our group showed the contemporary presence of MMTV sequences in tumor cell areas and their absence in the epithelial cells of normal glandular structures in material obtained by laser microdissection of the same histological slide. If endogenous, they would have been clearly present in both cell compartments, normal and tumoral.

## CONCLUSIONS

This paper confirms that MMTV*els* are present in a high percentage of SBCs, and shows them to be mostly absent in HBCs. Moreover, it demonstrates that MMTV-positive tumors test positive for the p14 protein, the signal peptide of the MMTV envelope precursor. Finally, the study favors the reliability of the data supporting the association between MMTV and sporadic breast carcinoma.

## METHODS

### Specimens

Forty-seven infiltrating ductal HBCs and 56 infiltrating ductal SBCs were analyzed for MMTV*els*. The formalin-fixed and paraffin-embedded (FFPE) samples, available for all 103 cases, were collected from the archive of the Department of Laboratory Medicine of the Pisa University Hospital. HBCs belonged to patients enrolled in the Pisa Center for Hereditary Tumors, selected based on the presence of a germline mutation affecting the BRCA1 gene or BRCA2 gene. SBCs belonged to patients older than 45 years with no family history for HBC.

### Laser microdissection

A Leica LMD automatic laser microdissector (Leica Microsystems, Wetzlar, Germany) was used to select the epithelial cell population to be studied. Sections (6 μm thick) were cut from each case using a new microtome blade for each slide, obtaining a total of 10,000 to 15,000 cells. Stromal and inflammatory cells were carefully excluded. This procedure was used for all the cases under study.

### DNA extraction

Microdissected samples were kept overnight in lysis buffer containing proteinase K (0.2 U per sample) obtained from Macherey-Nagel (Düren, Germany). Samples were processed for specific PCR amplification the next day. To avoid cross contamination, blank DNA samples (water) were processed in parallel with the tissue samples. We measured DNA concentration using Qubit 2.0 Fluorometer (Invitrogen, Life Technologies, Grand Island, NY) with the Qubit DNA HS assay kit. Moreover, to evaluate the quality of DNA we used the TapeStation (Agilent Technologies, Santa Clara, CA) with Genomic DNA Screen Tape.

### DNA amplification suitability

DNA was checked for the absence of PCR inhibitors by amplifying the human housekeeping GAPDH gene as the positive control.

### MMTV sequence detection and sequencing analysis

Fluorescence-nested PCR was used to detect the presence of the MMTV*els* sequence. Generated fluorescent amplicons were sized on an automatic DNA sequencer. Primer pairs were designed based on the sequence available in GenBank (accession no. AF243039). The outer primers yielded a 248-bp fragment from nucleotide positions 231 to 480 of MMTV*els*, and the inner primers yielded a 202-bp fragment (nucleotide positions 231 and 431). Sequences of the outer primers for the first PCR were as follows: forward, 5′-GATGGTATGAAGCAGGATGG-3′; and reverse, 5′-CCTCTTTTCTCTATATCTATTAGCTGAGGTAATC-3′. For the nested PCR, the forward primer sequence was the same as the one previously listed, whereas the reverse sequence was reverse nested (5′-AAGGGTAAGTAACACAGGCAGATGTA-3′). Both PCRs were performed in 50 μl containing 1X standard PCR buffer [1.5 mm MgCl_2_, 200 mM dNTP, 0.5 μM unlabeled reverse primer (MWG Biotech, Ebersberg, Germany), 0.5 μM 6-FAM–labeled forward primer (Applied Biosystems, Milan, Italy), and 2.5 U AmpliTaq Gold (ThermoFisher Scientific, Waltham, MA)]. The input target template was 500-ng genomic DNA in the first-round PCR and 2 μl of first-round PCR product in the second round. The amplification profile was as follows: one cycle at 94°C for 10 min; 40 (first-round) and 30 (second-round) cycles at 94 °C for 45 s, 58 °C for 45 s, and 72 °C for 60 s; and a final extension at 72 °C for 7 min. To exclude PCR contamination, water controls and negative DNA samples were included for every five samples in each run. Fluorescent amplicons were analyzed by capillary electrophoresis and appeared as peaks in an electropherogram. The amplicon size was extrapolated from a molecular size ladder resuspended in PCR buffer and run in parallel. Briefly, 3 μl of PCR products from both amplification rounds were mixed with 0.5 μl of ROX labeled size standard (Gene Scan 400 HD ROX; Applied Biosystems) and 11.5 μl of formamide (Hi-Di Formamide, Applied Biosystems). After denaturation at 95 °C for 3 min, samples were loaded onto an ABI PRISM 3130 XL automatic genetic analyzer and analyzed using GENESCAN software, version 3.1 (Applied Biosystems). The product of PCR amplification was sequenced, after clean up with the QIAquick PCR Purification Kit (Qiagen, Venlo, Netherlands), using Big Dye Terminator mix (Applied BioSystems). Sequencing reactions were run on an ABI PRISM 3130 XL (Applied BioSystems).

### Immunohistochemistry

Immunohistochemical assay was performed on 5 μm-thick paraffin sections. The antigen retrieval was achieved with MS-unmasker solution (DIAPATH, Martinengo, BG, Italy) in a microwave. Histostain–Plus kit (Invitrogen, Carlsbad, CA, USA) was used according to the manufacturer’s protocol. The slides were incubated with a primary antibody, rabbit polyclonal anti-MMTV-p14 (1:500 dilution), and then developed with diaminobenzidine chromogen (DAKO, Glostrup, Denmark) and counterstained with hematoxylin. Negative control included the omission of the primary antibody.

### Statistical analysis

To statistically analyze the distribution of MMTV*els*-positive and -negative samples in the two populations of infiltrating breast cancer samples (HBC and SBC), univariate analysis was performed using the two-tailed Fisher exact test. A value of *p* < 0.05 was considered statistically significant.

### Absence of contamination sources

The paraffin blocks were from the same archive and were processed in the same laboratory, which does not host mice nor murine cell cultures. On MMTV-positive samples, the presence of contaminating mouse DNA was excluded by performing murine mitochondrial DNA and IAP LTRs PCR [11], and murine GAPDH PCR.

### Ethics

Samples were collected anonymously according to the rules of the Ethics Committee of the Pisa University Hospital.
